# Dietary glycemic index, glycemic load, and renal cancer risk: findings from prostate, lung, colorectal, and ovarian cancer trial

**DOI:** 10.3389/fnut.2023.1073373

**Published:** 2023-06-06

**Authors:** Honggang Qi, Dan Xia, Xin Xu

**Affiliations:** Department of Urology, The First Affiliated Hospital, School of Medicine, Zhejiang University, Hangzhou, Zhejiang, China

**Keywords:** glycemic index, glycemic load, renal cancer, cohort, PLCO

## Abstract

**Background:**

Dietary glycemic index (GI) or glycemic load (GL) has been associated with the development of many cancers, but the evidence for renal cancer is still limited. The aim of the present study was to investigate the association between GI or GL and renal cancer risk in the Prostate, Lung, Colorectal, and Ovarian Cancer (PLCO) Screening Trial.

**Methods:**

The cohort for our analysis consisted of 101,190 participants. GI and GL were calculated from the FFQ data using previously published reference values. Multivariate-adjusted hazard ratios (HRs) and 95% confidence intervals (CIs) were estimated using Cox regression model after adjusting for most known renal cancer risk factors.

**Results:**

During a median of 12.2 years of follow-up, 443 incident renal cancer cases occurred. Higher dietary GI was significantly associated with a higher risk of renal cancer (HR_Q3vsQ1_: 1.38; 95% CI: 1.09–1.74; *p* for trend = 0.008). There was no significant association between dietary GL and renal cancer risk (HR_Q3vsQ1_ = 1.12, 95% CI = 0.79–1.59, *p* for trend = 0.591). Spline regression plot revealed a higher risk of renal cancer with a higher GI but not GL. There was no statistical evidence for nonlinearity (*p* for nonlinearity >0.05).

**Conclusion:**

In summary, findings of this large-scale prospective cohort study suggested that dietary GI may be associated with the risk of renal cancer. If confirmed in other populations and settings, dietary GI could be considered as a modifiable risk factor for renal cancer prevention.

## 1. Introduction

Renal cancer currently ranks seventh among the most frequently diagnosed cancer in males and ninth in females (1). In 2020, more than 431,000 cases of renal cancer were projected to occur worldwide (2). The geographic distribution of renal cancer is highest in the Baltic countries and in Eastern European countries and lowest in most parts of Asia and Latin America (3). There is a consistent male to female excess of renal cancer observed in both low- and high-incidence regions (4). The main established risk factors of renal cancer include tobacco smoking, body size, history of hypertension and chronic kidney disease (5, 6). However, they do not sufficiently explain these geographical and ethnic differences. Additional investigation is required to identify other suspected risk factors, which can improve prevention of renal cancer.

Emerging evidence have suggested a potential relationship between cancer development and diets associated with glucose and insulin metabolism. Hyperglycemia is associated with greater cancer risk and progression (7). This cancer-promoting effect may be mediated by systemic effects of insulin/insulin-related growth factor-1 (IGF-1) and inflammatory signaling. Direct uptake of glucose by cancer cells may also lead to epigenetic and biosynthetic changes (8). Glycemic index (GI) is a scale of zero to 100 for ranking carbohydrate-rich foods based on how quickly and how much they raise blood sugar levels after eating. Glycemic load (GL), a related measure, is a ranking system that takes into account the GI of a food and the carbohydrate content in a serving. High GI or GL has been associated with an increased risk of cardiovascular disease incidence and mortality (9–11) and some types of cancer (e.g., breast cancer) (12). A few prospective studies also have examined the potential association between dietary GI or GL and risk of renal cancer, with controversial results (13–15). A previous meta-analysis reported a significant positive association between GI and the risk of renal cancer (16). Given the potential impact of glucose and insulin on cancer, and limited evidence with mixed findings regarding GI and GL in relation to renal cancer risk, we investigated the associations of GI and GL with renal cancer risk within the large prospective Prostate, Lung, Colorectal and Ovarian (PLCO) Cancer Screening Trial.

## 2. Methods

### 2.1. Study population

PLCO screening trial was initially designed to evaluate whether screening tests might decrease mortality from prostate, lung, colorectal, and ovarian cancers. PLCO trial included 154,897 individuals aged 55 to 74 years at the inception of the study, recruited from 10 medical centers throughout the US from 1993 to 2001 (17). At enrollment, participants answered self-administered baseline questionnaire (BQ) and provided information on demographic information, medical history, anthropometric factors (i.e., height and weight), health behaviors, sex-associated exposures and other relevant factors. For the current analysis, 4,918 participants were excluded because of a lack of BQ data. We further excluded participants who did not complete a valid questionnaire or had history of cancer (*n* = 48,237), those had the highest or lowest 1 percentile of calorie intake (*n* = 546), and follow-up time was missing (*n* = 6). Ultimately, this resulted in the inclusion of 101,190 participants. The study protocol was approved by the institutional review boards of all participating centers, and all participants provided written informed consent (18). Our study was approved by the NCI with the project number of PLCO-1020.

### 2.2. Dietary assessment

The Diet History Questionnaire (DHQ) version 1.0 (National Cancer Institute, 2007) is a food frequency questionnaire that was added in 1998 and was administered to both arms of the trial. The DHQ collected a list of foods eaten in the past 12 months. The frequency and quantity of intake of 124 food items and supplement use were recorded (19). The DHQ has been found to do as well as or better than two widely used FFQs when the PLCO trial was conducted (19). The complete list of the GI and GL values for each food were based on the recent international tables (20) to find the optimal match as previously described (21). Weighted mean method was used to calculate the sex and serving size specific GLs for 225 food groups (19, 22). Each food’s GL was multiplied by the frequency of consumption of the food per day to calculate dietary GL for each participant. Daily GI was then calculated by dividing GL by total available carbohydrate intake and multiplying the result by 100. Total carbohydrates were classified as total available carbohydrates minus total dietary fiber.

### 2.3. Case ascertainment

Individuals were followed until cancer diagnosis or death, or end of follow-up (December 31, 2009). Participants were asked to update information about their health periodically with a self-administrated questionnaire. Participants were asked to identify whether and what type of cancer they had been diagnosed with in the previous year. The information of diagnosis date and location were also collected. Cancer registries, death certificates and physician reports also have been used as to provide additional data in cancer incidence. Medical record abstraction was performed by trained personnel to pathologically confirm all cancers. In this study, renal cancer case was defined as malignant neoplasm of renal parenchyma and renal pelvis coded using ICD-O-2 codes (C649 and C659).

### 2.4. Statistical analysis

The baseline information of the participants was presented by tertiles of dietary GI and GL. Hazard ratios (HR) with 95% confidence intervals (CIs) were evaluated using Cox proportional hazards regression models adjusted for the following potential confounders including age, sex, race, body mass index (BMI), education level, smoking status, drinking status, marital status, and total energy intake. We used Schoenfeld residuals to examine the proportionality of hazards (PH) assumption and no violation was found (23). To examine potential nonlinear associations, a restricted cubic spline model (24) with three knots (placed at the 10th, 50th, and 90th percentiles) was used to model a smooth curve. In sensitivity analysis, cancer cases occurring in the first two years of follow-up were excluded to minimize reverse causality. All statistical analyses were conducted using the software STATA version 15 (Stata Corp, College Station, TX, USA), with *p* < 0.05 considered statistically significant.

## 3. Results

### 3.1. Study characteristics

After a median of 12.2 years of follow-up, 443 incident renal cancer cases were identified. GI and GL from diet ranged from 32.97 to 79.16 (median value: 53.62) and from 9.97 to 494.89 (median value: 101.645), respectively. Participants with the highest GI or GL (i.e., tertile 3), compared with those with the lowest GI or GL (i.e., tertile 1), were more often men and current or former smokers. They also had higher total energy intake, were more likely to be black race, and had a higher BMI on average at baseline (Tables 1, 2).

**Table 1:** Main characteristic of participants in the PLCO cancer screening trial by GI.

Variables	Q1 (*n* = 33,763)	Q2 (*n* = 33,702)	Q3 (*n* = 33,725)	*p* value
Age (y), mean ± SD	62.4 (5.3)	62.5 (5.3)	62.3 (5.2)	< 0.001
Male (*n*, %)	14,529 (43.0%)	16,641 (49.4%)	17,909 (53.1%)	< 0.001
Arm (*n*, %)				< 0.001
Screen	17,469 (51.7%)	17,138 (50.9%)	16,924 (50.2%)	
Control	16,294 (48.3%)	16,564 (49.1%)	16,801 (49.8%)	
Smoking (*n*, %)				< 0.001
Never	16,626 (49.2%)	16,537 (49.1%)	15,202 (45.1%)	< 0.001
Current	2,745 (8.1%)	2,835 (8.4%)	3,741 (11.1%)	
Former	14,388 (42.6%)	14,323 (42.5%)	14,780 (43.8%)	
Missing	4 (<1%)	7 (<1%)	2 (<1%)	
Education (*n*, %)				< 0.001
≤High school	12,735 (37.7%)	13,716 (40.7%)	16,177 (48.0%)	
≥Some college	20,959 (62.1%)	19,923 (59.1%)	17,484 (51.8%)	
Missing	69 (0.2%)	63 (0.2%)	64 (0.2%)	
BMI (*n*, %)				< 0.001
<25.0 kg/m^2^	12,175 (36.1%)	11,250 (33.4%)	10,878 (32.3%)	
≥25.0 kg/m^2^	21,140 (62.6%)	22,021 (65.3%)	22,392 (66.4%)	
Missing	448 (1.3%)	431 (1.3%)	455 (1.3%)	
Race (*n*, %)				< 0.001
White, Non-Hispanic	31,532 (93.4%)	30,908 (91.7%)	29,611 (87.8%)	
Other	2,220 (6.6%)	2,782 (8.3%)	4,100 (12.2%)	
Missing	11 (<1%)	12 (<1%)	14 (<1%)	
Drinking (*n*, %)				<0.001
Never	3,430 (10.2%)	3,242 (9.6%)	3,413 (10.1%)	
Former	4,275 (12.7%)	4,542 (13.5%)	5,862 (17.4%)	
Current	25,194 (74.6%)	25,009 (74.2%)	23,362 (69.3%)	
Missing	864 (2.6%)	909 (2.7%)	1,088 (3.2%)	
Total energy intake (kcal/d), mean ± SD	1692.8 (704.3)	1736.0 (691.5)	1751.2 (714.8)	<0.001

GI, glycemic index; PLCO, prostate, lung, colorectal and ovarian; y, year; SD, Standard deviation; BMI, body mass index.

**Table 2:** Main characteristic of participants in the PLCO cancer screening trial by GL.

Variables	Q1 (*n* = 33,732)	Q2 (*n* = 33,735)	Q3 (*n* = 33,723)	*p* value
Age (y), mean ± SD	62.5 (5.3)	62.6 (5.3)	62.2 (5.3)	< 0.001
Male (*n*, %)	11,540 (34.2%)	15,654 (46.4%)	21,885 (64.9%)	< 0.001
Arm (*n*, %)				
Screen	17,263 (51.2%)	17,144 (50.8%)	17,124 (50.8%)	0.52
Control	16,469 (48.8%)	16,591 (49.2%)	16,599 (49.2%)	
Smoking (*n*, %)				< 0.001
Never	16,132 (47.8%)	16,398 (48.6%)	15,835 (47.0%)	
Current	3,187 (9.4%)	2,906 (8.6%)	3,228 (9.6%)	
Former	14,411 (42.7%)	14,424 (42.8%)	14,656 (43.5%)	
Missing	2 (<1%)	7 (<1%)	4 (<1%)	
Education (*n*, %)				< 0.001
≤ High school	14,507 (43.0%)	13,970 (41.4%)	14,151 (42.0%)	
≥ Some college	19,155 (56.8%)	19,694 (58.4%)	19,517 (57.9%)	
Missing	70 (0.2%)	71 (0.2%)	55 (0.2%)	
BMI (*n*, %)				< 0.001
< 25.0 kg/m^2^	11,843 (35.1%)	11,863 (35.2%)	10,597 (31.4%)	
≥ 25.0 kg/m^2^	21,436 (63.5%)	21,449 (63.6%)	22,668 (67.2%)	
Missing	453 (1.3%)	423 (1.3%)	458 (1.4%)	
Race (*n*, %)				< 0.001
White, Non-Hispanic	30,713 (91.1%)	30,959 (91.8%)	30,379 (90.1%)	
Other	3,005 (8.9%)	2,766 (8.2%)	3,331 (9.9%)	
Missing	14 (<1%)	10 (<1%)	13 (<1%)	
Drinking (*n*, %)				< 0.001
Never	3,445 (10.2%)	3,374 (10.0%)	3,266 (9.7%)	
Former	4,510 (13.4%)	4,764 (14.1%)	5,405 (16.0%)	
Current	24,756 (73.4%)	24,669 (73.1%)	24,140 (71.6%)	
Missing	1,021 (3.0%)	928 (2.8%)	912 (2.7%)	
Total energy intake (kcal/d), mean ± SD	1123.3 (332.0)	1633.5 (348.0)	2423.3 (630.8)	< 0.001

GL, glycemic load; PLCO, prostate, lung, colorectal and ovarian; y, year; SD, Standard deviation; BMI, body mass index.

### 3.2. Dietary GI or GL and renal cancer risk

As shown in Table 3, in categorical analysis with a maximally adjusted model, GI was significantly positively associated with renal cancer risk (HR_Q3vsQ1_: 1.38; 95% CI: 1.09–1.74; *p* for trend = 0.008). When GI was treated as a continuous variable, the HR (95% CI) of one-SD increment in the GI for renal cancer risk was 1.15 (1.04–1.26). HRs for renal cancer risk across GL tertiles are also presented in Table 3. After adjusting for various potential confounders, there was no significant association between GL and renal cancer risk (HR_Q3vsQ1_ = 1.12, 95% CI = 0.79–1.59, *p* for trend = 0.591). The results did not differ by continuous analysis. The HR (95% CI) of one-SD increment in the GL for renal cancer risk was 1.06 (0.89–1.28).

**Table 3:** Associations between GI/GL and renal cancer risk in the PLCO cancer screening trial.

Variables	Median	Cohort (*n*)	Cases (*n*)	Crude HR (95% CI)	Adjusted HR (95% CI)*
GI					
Q1 (< 52.29)	50.51	33,763	118	Reference group	Reference group
Q2 (≥ 52.29 to <54.94)	53.62	33,702	148	1.25 (0.98–1.59)	1.19 (0.93–1.51)
Q3 (≥ 54.94)	56.59	33,725	177	1.51 (1.20–1.91)	1.38 (1.09–1.74)
				*p* for trend <0.001	*p* for trend = 0.008
GL					
Q1 (< 85.06)	67.46	33,732	124	Reference group	Reference group
Q2 (≥ 85.06 to <120.26)	101.51	33,735	157	1.24 (0.98–1.57)	1.17 (0.91–1.51)
Q3 (≥ 120.26)	148.19	33,723	162	1.29 (1.02–1.63)	1.12 (0.79–1.59)
				*p* for trend = 0.045	*p* for trend = 0.591

*Adjusted for age (categorical), sex (male vs. female), race (non-Hispanic white vs. Other), body mass index (< 25 kg/m^2^ vs. ≥ 25 kg/m^2^), education (≤ high school vs. ≥ some college), smoking status (never vs. former vs. current), drinking status (never vs. former vs. current) and total energy intake (continuous). GI, glycemic index; GL, glycemic load.

### 3.3. Additional analyses

**Figure 1:**
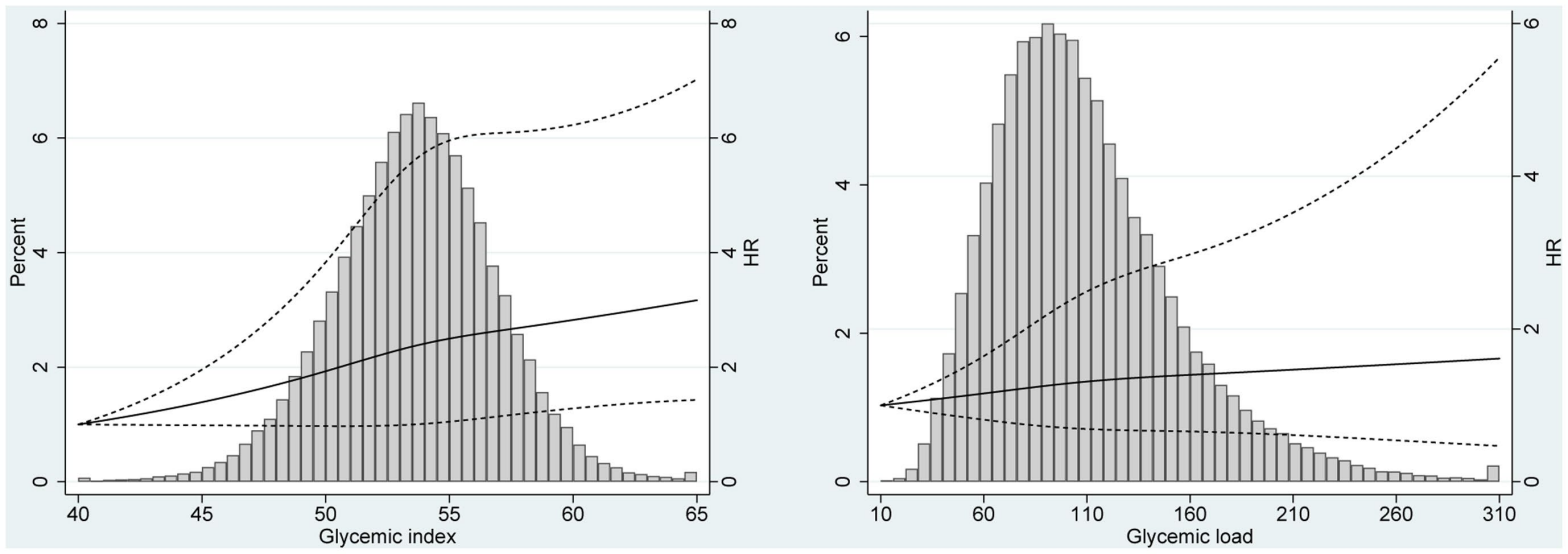
Spline regression plots of renal cancer risk in relation to **(A)** glycemic index (GI) and **(B)** glycemic load (GL). Hazard ratios (HRs) were calculated after adjusting for age (categorical), race (White, Non-Hispanic vs. Other), education (≤ high school vs. ≥ some college), smoking status (never vs. former vs. current), drinking status (never vs. former vs. current), body size (<25 kg/m^2^ vs. ≥25 kg/m^2^), and dietary energy intake (continuous). The histograms show the percentage of participants (left y axis) in each level of GI or GL.

The results of subgroup analyses based on several potential effect modifiers have been summarized in Tables 4, 5. The association between GI and renal cancer was more significant for studies conducted in male (HR_Q3vsQ1_ = 1.40, 95% CI = 1.04–1.88), in white non-Hispanic population (HR_Q3vsQ1_ = 1.51, 95% CI = 1.18–1.93) and in participants with BMI ≥ 25.0 kg/m^2^ (HR_Q3vsQ1_ = 1.45, 95% CI = 1.10–1.90). No significant associations were observed in any subgroups of the association between GL and renal cancer. Spline regression plots of renal cancer risk in relation to GI or GL are shown in Figure 1, which revealed a higher risk of renal cancer with a higher GI but not GL. There was no statistical evidence for nonlinearity (*p* for nonlinearity >0.05). In sensitivity analysis, there was little change in the findings after excluding cases who were diagnosed within the first two years of follow-up (GI: HR_Q3vsQ1_: 1.38; 95% CI: 1.09–1.76; GL: HR_Q3vsQ1_ = 1.11, 95% CI = 0.78–1.58).

**Table 4:** Subgroup analyses between GI and renal cancer risk.

Variables	Q1	Q2	Q3	*p* for interaction
Sex				> 0.05
Male	Reference	1.19 (0.87–1.61)	1.40 (1.04–1.88)	
Female	Reference	1.18 (0.80–1.75)	1.32 (0.89–1.95)	
Race				< 0.05
White, Non-Hispanic	Reference	1.20 (0.93–1.54)	1.51 (1.18–1.93)	
Other	Reference	1.09 (0.50–2.38)	0.48 (0.20–1.14)	
BMI (*n*, %)				> 0.05
<25.0 kg/m^2^	Reference	0.97 (0.60–1.58)	1.19 (0.75–1.89)	
≥25.0 kg/m^2^	Reference	1.26 (0.95–1.67)	1.45 (1.10–1.90)	

GI, glycemic index; BMI, body mass index.

**Table 5:** Subgroup analyses between GL and renal cancer risk.

Variables	Q1	Q2	Q3	*p* for interaction
Sex				> 0.05
Male	Reference	1.18 (0.84–1.66)	1.14 (0.74–1.76)	
Female	Reference	1.11 (0.73–1.70)	1.00 (0.51–1.95)	
Race				> 0.05
White, Non-Hispanic	Reference	1.19 (0.91–1.56)	1.22 (0.84–1.76)	
Other	Reference	0.98 (0.42–2.25)	0.37 (0.10–1.42)	
BMI (*n*, %)				> 0.05
<25.0 kg/m^2^	Reference	1.14 (0.68–1.89)	0.78 (0.37–1.62)	
≥25.0 kg/m^2^	Reference	1.17 (0.87–1.59)	1.25 (0.84–1.88)	

GL, glycemic load; BMI, body mass index.

## 4. Discussion

In this large prospective PLCO cohort, there was a statistically significant association between dietary GI and renal cancer risk. No obvious association between GL and renal cancer risk was observed. Similar results were obtained when excluding cases diagnosed within the first two years of follow-up. Findings from continuous analyses and spline regression plots were comparable with the results in main analyses.

These results, which were based on a large prospective cohort study, were consistent with our previous findings based on a meta-analysis (16). A significant positive association was observed between GI and the risk of renal cancer (poled RR 1.16, 95% CI 1.02–1.32). GL was not significantly associated with renal cancer risk (pooled RR 1.14, 95% CI 0.81–1.60). However, only five studies (three case–control and two cohort studies) were eligible in this meta-analysis. Case–control studies were more likely to be prone to recall bias and select bias. The evidence on the association between GI or GL and renal cancer risk may be still limited and not robust. Therefore, we further performed an analysis based on a large US cohort. As a result, we also found a significant association between GI and renal cancer risk and no association was observed for GL in PLCO cohort, which further enhanced the current evidence.

In subgroup analyses for GI, a more significant association was observed in male, in white non-Hispanic population and in overweight/obese participants, which suggested a potential differential susceptibility. The incidence of renal cancer has obvious ethnic and sex differences. In addition. A high BMI is also a well-established risk factor for renal cancer.

Several mechanisms have been proposed to explain a potential association between dietary GI and human cancers. Foods with a high GI will increase the concentration of glucose and insulin in blood and thus induce hyperinsulinemia (25, 26). Hyperinsulinemia can increase the insulin-like growth factor-1 (IGF-1) expression (27). Higher IGF-1 has been reported to be modestly associated with increased risk of overall cancer risk, including kidney cancer based on a cohort study analysis from the UK Biobank (28). Previous studies have demonstrated that IGF-1 pathway plays an important role in cell proliferation and apoptosis resistance in renal cell carcinoma (29). In addition, long-term exposure of tubular cells to hyperglycemia can lead to disturbances in DNA repair mechanisms, which may drive and promote renal cancer development (30).

Several limitations of the current study should be acknowledged. First, the outcome of this study was renal cancer incidence. Although we have excluded the renal pelvis cancer, we were not able to further classify the types of renal cancer because of the limited data on original questionnaire. Second, the majority of GI values (32.97 to 79.16, median value: 53.62) centered around the middle of the theoretical range for GI (i.e., 0–100) in PLCO trial. Therefore, it was hard to evaluate the effects of different levels of GI unless it is a strong cancer risk determinant at middle values (31). Third, dietary questionnaire used in PLCO cohort was self-administrated, which may cause some measurement error (32). In addition, dietary intake was assessed only once at baseline and any changes in diet during follow-up could not be examined. Finally, although the statistical models were adjusted for various important confounders, a certain degree of residual confounding may be unavoidable because of collinearity from other nutrients, particularly macronutrients.

This study had some unique strengths. As a prospective study, the chance of reverse causality from subclinical disease-causing changes in diet was small and the recall bias was avoided as the exposure was preceding the onset of cancer. This study included almost 100,000 participants with a median of 12.2 years of follow-up, which provided strong power to detect differences in renal cancer incidence if they truly existed. Additionally, the rate of participants lost to follow up was very low in PLCO study. The large study population was recruited from institutions across the United States, which improved the generalizability. The availability of data on various potential confounders made comprehensive adjustment possible. Lastly the methods used to assign GI and GL values to foods was rigorous, which was based on American data wherever possible (21).

In summary, analysis of the PLCO cohort suggested that diets high in GI was associated with greater renal cancer risk. If confirmed in other populations and settings, dietary GI could be considered as a modifiable risk factor for renal cancer prevention.

## Data availability statement

The datasets presented in this study can be found in online repositories. The names of the repository/repositories and accession number(s) can be found at: https://cdas.cancer.gov/datasets/plco/.

## Ethics statement

The studies involving human participants were reviewed and approved by National Cancer Institute. The patients/participants provided their written informed consent to participate in this study.

## Author contributions

XX and DX contributed to the conception or design of the work. XX, HQ, and DX contributed to the acquisition, analysis, or interpretation of data for the work. HQ and DX drafted the manuscript. XX critically revised the manuscript. All authors gave final approval and agreed to be accountable for all aspects of work ensuring integrity and accuracy.

## Funding

This study was supported by grants from the Zhejiang Provincial Natural Science Foundation (LY22H160027) and National Natural Science Foundation of China (82172597).

## Conflict of interest

The authors declare that the research was conducted in the absence of any commercial or financial relationships that could be construed as a potential conflict of interest.

## Publisher’s note

All claims expressed in this article are solely those of the authors and do not necessarily represent those of their affiliated organizations, or those of the publisher, the editors and the reviewers. Any product that may be evaluated in this article, or claim that may be made by its manufacturer, is not guaranteed or endorsed by the publisher.
